# Evaluation of the Immunomodulatory Activity of the Chicken NK-Lysin-Derived Peptide cNK-2

**DOI:** 10.1038/srep45099

**Published:** 2017-03-23

**Authors:** Woo H. Kim, Hyun S. Lillehoj, Wongi Min

**Affiliations:** 1 Animal Biosciences and Biotechnology Laboratory, Beltsville Agricultural Research Center, ARS, U.S. Department of Agriculture, Beltsville, MD 20705, USA; 2 College of Veterinary Medicine & Institute of Animal Medicine, Gyeongsang National University, Jinju 52828, Korea

## Abstract

Chicken NK-lysin (cNK-lysin), the chicken homologue of human granulysin, is a cationic amphiphilic antimicrobial peptide (AMP) that is produced by cytotoxic T cells and natural killer cells. We previously demonstrated that cNK-lysin and cNK-2, a synthetic peptide incorporating the core α-helical region of cNK-lysin, have antimicrobial activity against apicomplexan parasites such as *Eimeria* spp., via membrane disruption. In addition to the antimicrobial activity of AMPs, the immunomodulatory activity of AMPs mediated by their interactions with host cells is increasingly recognized. Thus, in this study, we investigated whether cNK-lysin derived peptides modulate the immune response in the chicken macrophage cell line HD11 and in chicken primary monocytes by evaluating the induction of chemokines, anti-inflammatory properties, and activation of signalling pathways. cNK-2 induced the expression of CCL4, CCL5 and interleukin(IL)-1β in HD11 cells and CCL4 and CCL5 in primary monocytes. We also determined that cNK-2 suppresses the lipopolysaccharide-induced inflammatory response by abrogating IL-1β expression. The immunomodulatory activity of cNK-2 involves the mitogen-activated protein kinases-mediated signalling pathway, including p38, extracellular signal-regulated kinase 1/2 and c-Jun N-terminal kinases, as well as the internalization of cNK-2 into the cells. These results indicate that cNK-2 is a potential novel immunomodulating agent rather than an antimicrobial agent.

With the increasing emergence of antibiotic-resistant pathogens, antimicrobial peptides (AMPs) have been studied as alternatives to antibiotics based on their broad spectrum of bactericidal activity and selectivity^1^. Cationic antimicrobial peptides are highly conserved in all organisms and are effective against many bacteria, including multidrug-resistant bacterial strains, by disrupting the bacterial membrane based on their cationic nature^2^. However, the direct activity of cationic AMPs towards the microbial membrane is dependent on physiological conditions, such as salt and serum^3^. For example, the antimicrobial activity of LL-37, a human cathelicidin, against *Salmonella* spp. is abolished in the presence of tissue-culture medium^4^. Increasing evidence indicates that direct microbial killing may not be the primary role of cationic AMPs in the body, and efforts to determine the true role of cationic AMPs have focused on the immunomodulatory properties of cationic AMPs^5^. The immunomodulatory activity of cationic AMPs is complex and includes anti-infective immune modulation, such as the induction of chemokines and cytokines, pro/anti-inflammatory activity, direct chemotaxis, wound healing, angiogenesis, apoptotic activity and adjuvant activity^6^,^7^,^8^. The immunomodulatory activity of cationic AMPs also varies depending on the cell type. Because of their ability to modulate the immune response, it has been proposed that cationic AMPs be called host defence peptides (HDPs). HDPs have been studied extensively in mouse models, but there have been few studies of avian peptides.

Chicken NK-lysin (cNK-lysin) is a homologue of human granulysin. Human granulysin is found in the cytolytic granules located in human natural killer (NK) and cytotoxic T lymphocytes (CTLs)^9^. We previously demonstrated that cNK-lysin is highly expressed in *Eimeria*-infected intestinal lymphocytes, suggesting a role in parasite infection^10^. Subsequent studies have shown that cNK-lysin and cNK-2, the core α-helical region of cNK-lysin, can kill *Eimeria* sporozoites by disrupting the parasitic membrane. Interestingly, cNK-2 exhibits higher antimicrobial activity than the original peptide and even melittin, a powerful non-specific AMP from honeybees, indicating that the modification of the natural sequence can improve efficiency. Among four synthetic cNK-lysin derived peptides, only cNK-2 is effective, which may reflect the incorporation of the core α-helical region in its structure. A protective effect of administration of cNK-2 *in ovo* and intraperitoneally against *Eimeria* infection was subsequently identified in *E. acervulina*-infected chickens. The administration of cNK-2 induced a significant increase in body weight and reduced gut-lesion scores^11^,^12^. However, the mechanisms underlying the immune response to cNK-lysin peptides remain unknown.

Granulysin acts as an immunomodulatory peptide by serving as a chemoattractant for lymphocytes and modulating the expression of chemokines and cytokines^13^,^14^. In this study, we demonstrated that cNK-2 has immunomodulatory properties as an HDP, including inducing chemokines/cytokines, an anti-inflammatory response, signalling pathway activation and internalization into cells. By contrast, the antimicrobial effects of cNK-2 were reduced under physiological salt conditions. The responses of HD11 cells and primary monocytes to cNK-2 were also compared to understand the role of cNK-2 in innate immunity. Not all of our findings are consistent with previously studied cationic AMPs or HDPs, and this study provides advanced insight on how the chicken immune response is modulated by peptides.

## Results

### Antimicrobial activity of the cNK-lysin peptide in the presence of salts

**Figure 1:**
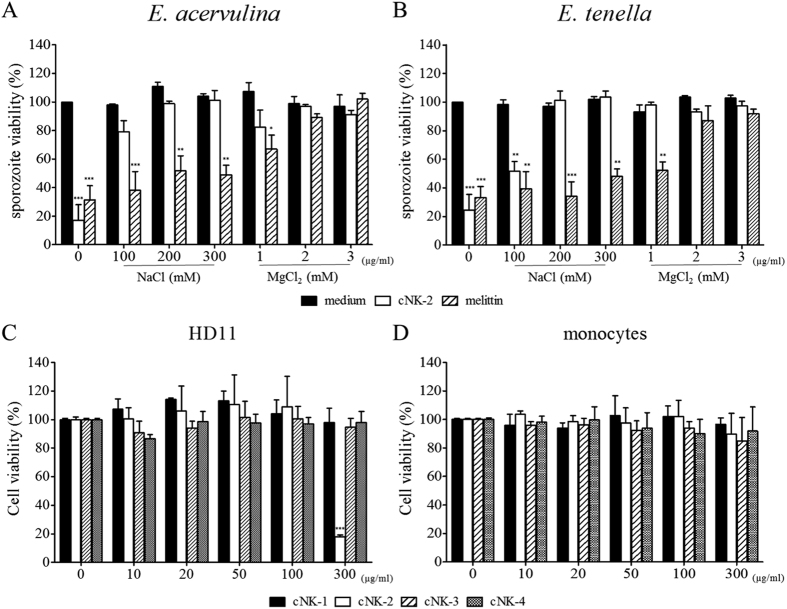
Antimicrobial activity in the presence of salts and cytotoxicity of cNK-lysin peptides against chicken cells. The antimicrobial activity against *E. acervulina* (**A**) and *E. tenella* (**B**) was determined by counting viable sporozoites after a 6 h incubation with cNK-lysin peptides or melittin, as a positive control, in the presence of NaCl or MgCl_2_. Cytotoxicity was determined by the CCK-8 assay after a 24 h stimulation with cNK-lysin peptides (0–300 μg/ml) in HD11 cells (**C**) and primary monocytes (**D**). The data represent the average of three independent experiments ± SE. *p *<* *0.001 (***) was considered statistically significant compared to the medium control.

The antimicrobial effect of the cNK-lysin peptides against *Eimeria* spp. was determined in the presence of NaCl and MgCl_2_ by viable counting of sporozoites by trypan blue exclusion. Consistent with a previous report^12^, cNK-2 exhibited a high antimicrobial effect against *Eimeria* sporozoites. However, the antimicrobial effect of cNK-2 was antagonized by salts, and the viabilities of *E. acervulina* and *E. tenella* were nearly recovered in the presence of 200 mM NaCl or 2 mM MgCl_2_ (Fig. 1A and B). This result indicated that the direct antimicrobial activity of cNK-2 might not be the most relevant function of cNK-2. Melittin, which was used as a positive control, exhibited greater resistance to salts than cNK-2. By contrast, up to 10% FBS had no effect on the antimicrobial effects of cNK-2 (data not shown).

### Cytotoxicity of cNK-lysin peptides in chicken cells

Prior to studying the effect of the peptides in an *in vitro* cell system, the cytotoxicity of the peptides against the target cells should be investigated. Thus, we next assessed the cytotoxic effect of cNK-2 in chicken cells, HD11 cells and primary monocytes. The cells were treated with different concentrations of the cNK-2 peptides for 24 h, and the viability was determined by CCK-8. There was no significant reduction in cell viability at concentrations of ≤100 μg/ml, whereas cNK-2 had a significant cytotoxic effect on HD11 cells but not monocytes at a concentration of 300 μg/ml (Fig. 1C and D).

### cNK-2-induced expression of chemokines and cytokines in chicken cells

**Figure 2:**
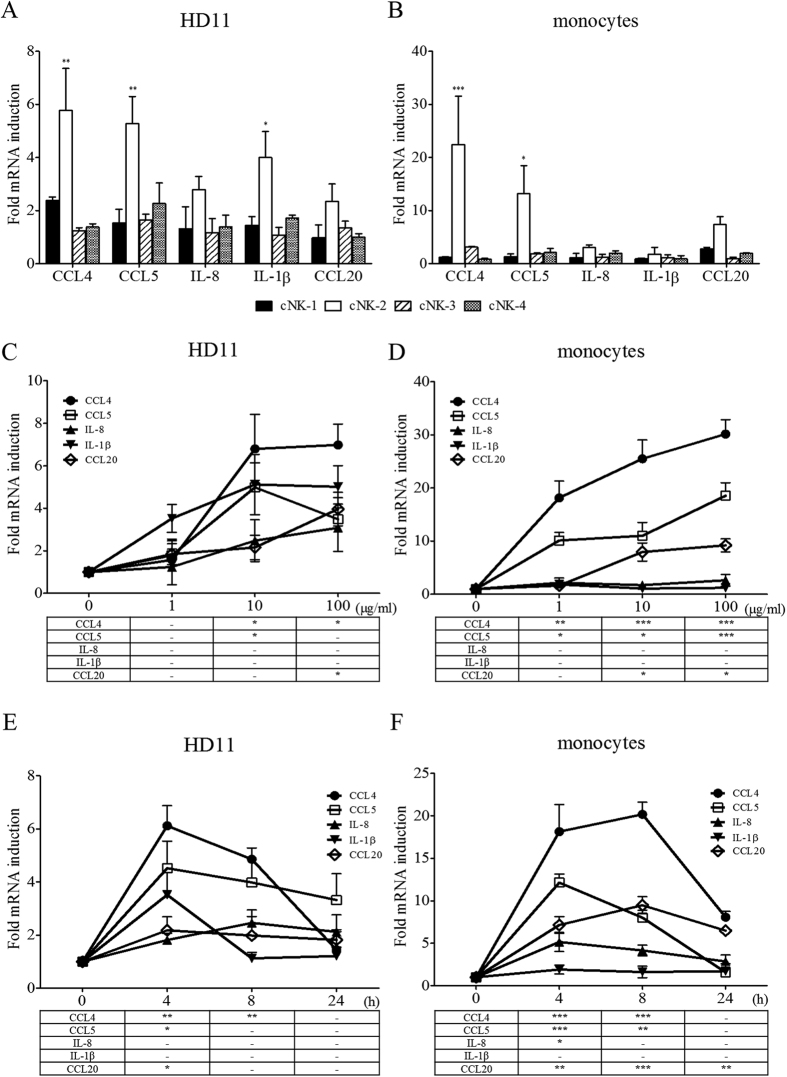
Stimulation of innate immunity by cNK-lysin peptides. HD11 cells (**A**) and primary monocytes (**B**) were stimulated with 10 μg/ml cNK-lysin peptides for 4 h. RNA was isolated and used for real-time qPCR in triplicate. The fold changes in the expression of each gene were normalized to β–actin and are relative to the gene expression in unstimulated cells. The kinetics of the cNK-2-induced responses were dose-dependent (**C** and **D**) and time-dependent (**E** and **F**). The data represent the mean ± SE from two or three independent experiments. *p *<* *0.01 (**) and *p *<* *0.001 (***) were considered statistically significant compared to the medium control.

The ability of cNK-lysin peptides to modulate the immune response was determined by measuring the expression of several chemokines and cytokines. Prior to investigate the effect of cNK-2 peptides, the ability of full-length cNK-lysin to modulate immune response was determined. However, there was no chemokines that induced by cNK-2 in HD11 and monocytes (see Supplementary Fig. S1). Interestingly, it has revealed that only cNK-2 but not the other cNK-lysin peptides induced several chemokines and cytokines after 4 h of stimulation with the peptides (Fig. 2A and B). CCL4, CCL5 and interleukin-1β (IL-1β) were significantly upregulated in HD11 cells, and CCL4 and CCL5 were upregulated in the primary monocytes. The primary monocytes exhibited stronger responses: CCL4 and CCL5 were upregulated 22.5-fold and 13.2-fold, respectively, in primary monocytes but 5.3-fold and 5.8-fold in HD11 cells. The induction of chemokines and cytokines by cNK-2 was dose- but not time-dependent as it decreased mostly at 24 h (Fig. 2C–F). The chemokine and cytokine genes induced by cNK-2 peaked at 4 h after stimulation in both cell types tested. Although the expression patterns of chemokines and cytokines were similar between HD11 cells and primary monocytes, there was a notable difference in the expression of IL-1β: HD11 cells exhibited significantly increased expression, whereas IL-1β was not induced in primary monocytes. It also has found that the protein levels of chemokines measure by ELISA are consistent with mRNA expression (see Supplementary Fig. S2). Collectively, these results suggest immunomodulatory roles of cNK-2 in macrophages and monocytes and that it might be differentially modulated in different cell types.

### cNK-2 suppresses LPS-induced inflammatory cytokine expression

**Figure 3:**
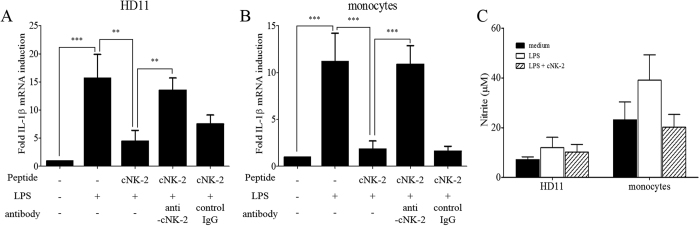
Modulation of the LPS-induced pro-inflammatory responses by cNK-2. HD11 cells (**A**) and primary monocytes (**B**) were incubated with 10 μg/ml cNK-2 and 1 μg/ml *E. coli* LPS. RNA was isolated after 4 h and used for real-time qPCR in triplicate. (**C**) The induction of NO production by *E. coli* LPS alone or in the presence of cNK-2 was determined by a Griess assay. The data represent the mean ± SE from two or three independent experiments. *p *<* *0.01 (**) and *p *<* *0.001 (***) were considered statistically significant compared to the medium control.

To evaluate a possible role in the inflammatory response, we investigated whether cNK-2 suppresses the expression of inflammatory cytokines induced by LPS in chicken cells. HD11 cells and monocytes were stimulated with LPS in the presence or absence of cNK-2. cNK-2 significantly suppressed the expression of the pro-inflammatory cytokine IL-1β by 71.1% in HD11 cells and 83.2% in primary monocytes (Fig. 3A and B). The reduction of IL-1β by cNK-2 was restored by a rabbit anti-cNK-2 antibody, suggesting cNK-2 specific response confirmed by neutralization of cNK-2 antibody (Fig. 3A and B). Because increased expression of IL-1β might trigger nitric oxide (NO) production in macrophages, we also investigated whether LPS and cNK-2 treatment induced NO production. As shown Fig. 3C, cNK-2 reduced the production of NO induced by LPS, but this change was not significant in either cell type.

### Signalling pathways mediate the chemokine-induction activity of cNK-2

**Figure 4:**
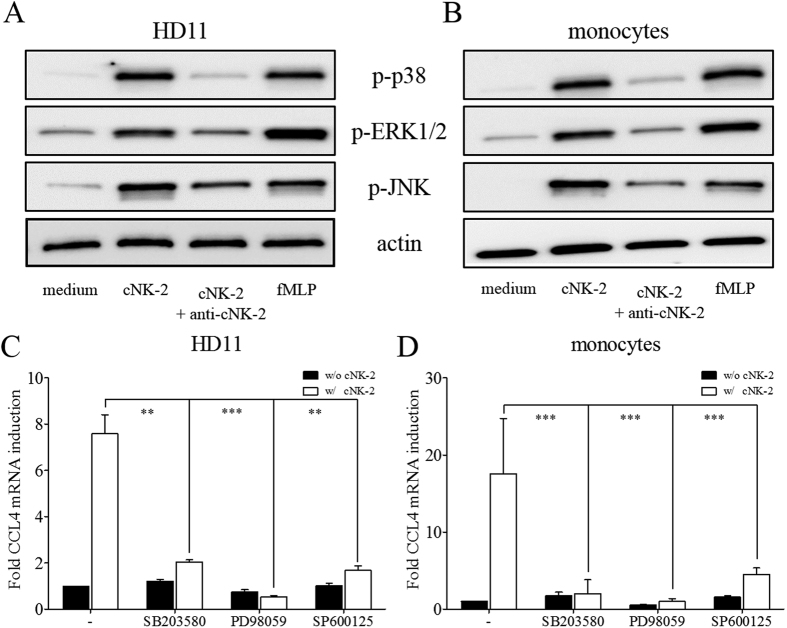
Activation of the MAPK pathway by cNK-2. HD11 cells (**A**) and primary monocytes (**B**) were stimulated with 10 μg/ml cNK-2 or fMLP as a positive control for 30 min. The levels of phosphorylated p38 (p-p38), phosphorylated ERK1/2 (p-ERK1/2), phosphorylated JNK (p-JNK) and β-actin in the cellular lysates were determined by Western blot analysis. The effects of MAPK inhibitors (10 μM each) on p38 (SB203580), ERK1/2 (PD98059) and JNK (SP600125) in HD11 cells (**C**) and primary monocytes (**D**) were determined by real-time qPCR. *p* < 0.01 (**) and *p* < 0.001 (***) were considered statistically significant compared to the vehicle control.

Some host defence peptides, such as LL-37 and IDR peptides, stimulate chemokine production through the mitogen-activated protein kinases (MAPK) pathway^15^,^16^,^17^, and thus we determined which MAPK signalling pathways are activated by cNK-2. In the presence of cNK-2, the phosphorylation of p38, ERK and JNK was induced in HD11 cells and primary monocytes with similar patterns (Fig. 4A and B). To investigate the relationship between the activation of the MAPK pathways and chemokine induction, specific inhibitors of the MAPK pathway were used. As shown in Fig. 4C and D, the expression of CCL4 was significantly abrogated in the presence of inhibitors, including SB203580, PD980059 and SP600125 (p38, ERK1/2 and JNK inhibitors, respectively), suggesting that activation of the MAPK pathways is not only induced by cNK-2 stimulation but is also necessary for the cNK-2-induced immune response.

### Internalization of cNK-2

**Figure 5:**
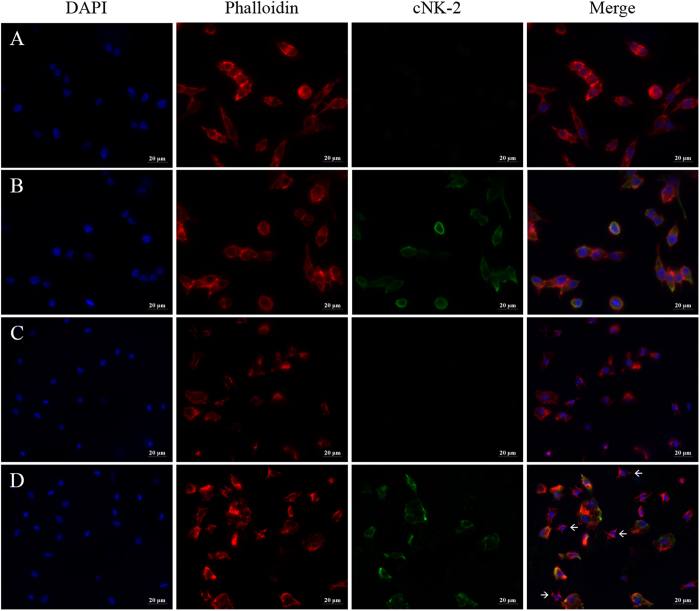
Cellular localization of cNK-2. HD11 cells (**A** and **B**) and primary monocytes (**C** and **D**) were incubated with medium alone (**A** and **C**) or 10 μg/ml cNK-2 (**B** and **D**) for 30 min at 41 °C. Immunocytochemistry was performed with a rabbit polyclonal cNK-2 (green) antibody followed by an Alexa Fluor 488 goat anti-rabbit IgG secondary antibody. DAPI and Alexa Fluor 555 Phalloidin were used to stain nuclei (blue) and F-actin (red), respectively. The scale bar in all figures corresponds to 20 μm.

**Figure 6:**
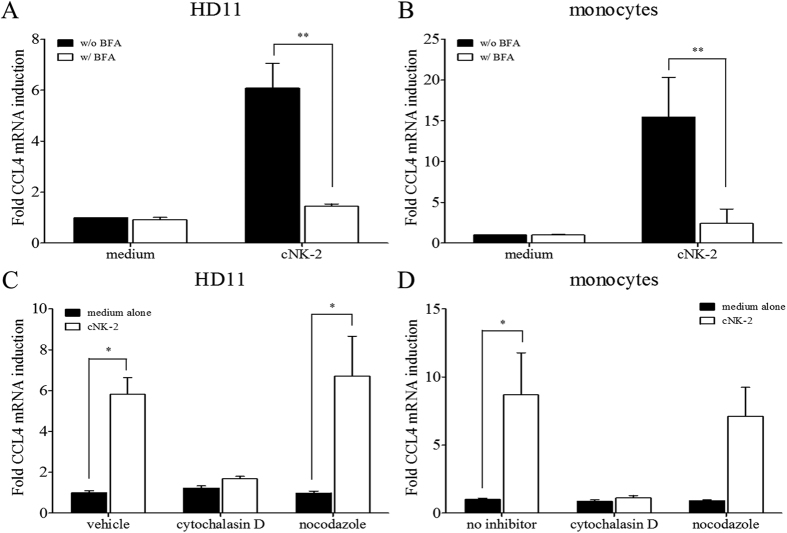
Effect of endocytosis inhibitors on the expression of chemokines induced by cNK-2. HD11 cells (**A** and **C**) and primary monocytes (**B** and **D**) were incubated with 10 μg/ml cNK-2 in the presence or absence of 5 μg/ml Brefeldin A, 5 μg/ml cytochalasin D, or 5 μg/m nocodazole. The cells were pre-treated with Brefeldin A (**A** and **B**), cytochalasin D or nocodazole (**C** and **D**) for 1 h prior to incubation with cNK-2 for 4 h. The data represent the mean ± SE from two independent experiments. *p *<* *0.05 (*) and *p *<* *0.01 (**) were considered statistically significant compared to the medium control. BFA, Brefeldin A.

Because cNK-2 induces an immune response that upregulates chemokines and stimulates the activation of signalling pathways, we hypothesized that a molecular interaction occurs between cNK-2 and the target cells. Thus, we investigated whether cNK-2 is taken up into the cells using a polyclonal cNK-2 antibody. As shown in Fig. 5, intracellular cNK-2 was localized throughout the cytoplasmic region of HD11 cells and the primary monocytes at 30 min after stimulation. Interestingly, some primary monocytes were not stained by the cNK-2 antibody, whereas nearly all HD11 cells were positive for cNK-2 staining. The population of cNK-2-negative cells (indicated by the arrows) was smaller and less differentiated than the cNK-2-positive cells (see Supplementary Fig. S3 for better images processed by confocal microscope). Although we could not conclude whether the morphological changes of the cells were caused by cNK-2 stimulation or were due to normal characteristics of the primary monocyte population, this observation suggests that cNK-2 is selectively internalized into cells. Moreover, we evaluated the ability of endocytosis to modulate the immune response using several endocytosis inhibitor, Brefeldin A as a general endocytosis inhibitor, cytochalasin D as an inhibitor for actin polymerization and nocodazole as an inhibitor for microtubule polymerization. The induction of the expression of CCL4 by cNK-2 was suppressed in Brefeldin A-treated HD11 cells and primary monocytes, suggesting that endocytosis of cNK-2 is required for modulating the immune response (Fig. 6A and B). As shown in Fig. 6C and D, the induction of CCL4 by cNK-2 was inhibited by cytochalasin D in but not by nocodazole in both cells suggesting that the actin polymerization is related with endocytosis of cNK-2.

## Discussion

Traditionally, studies of cationic AMPs have focused on their antimicrobial functions against diverse microorganisms as part of efforts to develop alternative treatments for multidrug-resistant bacteria^18^. We previously noted the antimicrobial activity of cNK-lysin as well as its derivatives^11^,^12^ and demonstrated that cNK-lysin peptides are among the few cationic peptides that kill not only bacteria but also parasites through a direct interaction that results in disruption of the plasma membrane. Unfortunately, the direct binding of peptides to the microbial membrane is severely compromised under physiological conditions, including high monovalent or moderate divalent cation concentrations, host proteases, polyvalent anions and serum, which indicates that the direct killing of microbes might not be a major function of cationic peptides *in vivo*^4^,^19^. The reduction of the antimicrobial activity of cationic AMPs under physiological conditions has been attributed to their overall positive charge, which ensures accumulation at the polyanionic microbial cell surface containing acidic polymers such as LPS and teichoic acids^20^. In present study, the antimicrobial activity of cNK-2 against *Eimeria* spp. was abrogated in medium containing physiologically relevant concentrations of salts (200 mM NaCl or 2 mM MgCl_2_), but not in the presence of serum as constant medium supplemented with 10% FBS was used throughout the immunomodulation studies. Cationic peptides are inactivated by body fluids containing certain level of salts due to the binding of blood components, protease degradation or competition for membrane binding sites^21^ and it suggests that certain components bind to the surface membrane of sporozoites, interrupting the interaction of cNK-2 with binding sites; *in vivo*, cNK-2 likely functions by both directly killing parasite sporozoites and modulating the immune response.

In recent years, there has been increased effort to investigate the interaction of cationic peptides with host cells as immunomodulators in mammals^17^,^22^,^23^,^24^, but these peptides remain poorly studied in avian species. In this study, we demonstrated the potential of the cNK-lysin derived peptide cNK-2 as an immunomodulating agent in terms of the induction of chemokine and cytokine production, modulation of the TLR agonist-induced inflammatory response, activation of signalling pathways and interactions with target cells. Because macrophages and monocytes are believed to participate in various innate immune responses induced by cationic peptides^25^,^26^, the chicken macrophage cell line HD11 and primary monocytes derived from PBMCs were used for this study. In addition, a rabbit polyclonal antibody against cNK-2 was developed and used to confirm the specificity of the cNK-2 activities by neutralization throughout this study. Of the four cNK-lysin derived peptides, cNK-2 was the only peptide to exhibit immunomodulating activities. cNK-2 induced chemokine and cytokine expression at a concentration range of 1 to 100 μg/ml which is a wider range than doses displaying antimicrobial activity. LL-37 and several innate defence regulator (IDR) peptides induce chemokine production in a variety of cells, including PBMCs, dendritic cells, neutrophils, monocytes and macrophages^15^,^16^,^24^,^27^. Because the chemokines and cytokines induced by different immunomodulatory peptides vary and overlap, analysing the expression profiles produced by these peptides is important to understand their effects. The majority of chemokines induced by cationic peptides in mammals are inflammatory chemokines, such as MCP-1 (CCL2), MIP-1α (CCL3), MIP-1β (CCL4), RANTES (CCL5), MCP-3 (CCL7), Gro-α (CXCL1) and IL-8 (CXCL-8), which are produced at high concentrations during infection, suggesting that cationic peptides modulate the inflammatory response induced by diverse pathogens^28^. Unfortunately, the number of available chemokines and cytokines in avian species is not known^29^, and therefore we were unable to assess a variety of chemokine and cytokine panels. However, our results demonstrated that the inflammatory chemokines in chickens, CCL4 and CCL5, were induced by cNK-2 in innate immune cells, suggesting a role of this peptide in association with inflammatory response-mediated infections. CCL4 shares the chemokine receptor CCR5 with CCL5 for its chemotactic function^30^, indicating that this receptor might be among the target molecules used to modulate the cNK-2 induced immune response. Indeed, it has been reported that IDR-1002 induced monocyte chemotaxis regulated through the CCR5 and its ligands CCL3 and CCL5^31^.

Next, we demonstrated that cNK-2 modulates the inflammatory response induced by LPS, with reduced expression of the pro-inflammatory cytokine IL-1β in HD11 cells and monocytes. This observation is consistent with a previous result showing that chicken cathelicidin-2 (CATH-2) and its analogues neutralize LPS-induced IL-1β expression in HD11 cells^32^. Many cationic peptides inhibit pro-inflammatory responses, such as those produced by several Toll-like receptor ligands, including LPS and lipoteichoic acid, by reducing pro-inflammatory mediators (mostly TNF-α)^24^,^33^. Notably, LPS-induced inflammatory responses were inhibited by approximately 50% by cNK-2 in both macrophages and monocytes, and no significant inhibition of NO production was observed, whereas CATH-2 entirely abolished IL-1β expression and significantly neutralized NO production induced by LPS^32^. These data indicate that either the ability of cNK-2 to inhibit inflammatory responses is weaker than that of CATH-2 and its analogues or that the mechanism by which cNK-2 modulates the inflammatory responses induced by LPS differs from that used by CATH-2.

To understand the cellular mechanisms underlying the responses induced by cNK-2, the profile of the MAPK family members activated by cNK-2 in chicken cells was investigated. Western blot analysis revealed the involvement of at least three types of MAPK signalling pathways in the cNK-2-induced immune response: p38, ERK1/2 and JNK. These proteins are the main components of the pathway and play a critical role in a variety of cellular activities, including the expression of chemokines^34^. The activation of these MAPK pathways by cationic peptides is in agreement with previous studies using LL-37 and IDR-1002 in mice cells, which demonstrated that these peptides signal through the MAPK pathways to modulate the innate immune response^15^,^16^. The activation of signalling pathways followed by the binding of an intracellular target with cationic peptides is an important consideration in the evaluation of immunomodulatory properties, particularly in the inflammatory process because several cationic peptides suppress inflammatory responses through well-known signalling pathways, such as the MAPK signalling pathway^35^. The use of specific pathway inhibitors further confirmed that the activation of p38, ERK1/2, and JNK is required for cNK-2-induced chemokine expression because the p38 inhibitor SB203580, the ERK1/2 inhibitor U0126 or the JNK inhibitor SP600125 abolished the induction of CCL4 and CCL5. This study is the first to directly examine the roles of MAPK in chicken cells treated with a chicken-derived cationic peptide.

Generally, modulation of immune responses, such as chemokine induction, by cationic peptides requires cellular uptake^26^,^36^. Some cell surface or intracellular molecules, including formyl peptide receptor-like 1 (FPRL1), GAPDH, p62/SQSTM1 and G_i_-coupled receptor, have been described as receptors for cationic peptides, although the exact mechanism of the receptor interaction has not been elucidated^25^,^26^,^37^,^38^. Although we could not identify the interacting protein partners of cNK-2, we observed cellular uptake in a Brefeldin A-dependent manner and intracellular localization of cNK-2, suggesting a possible interaction with intracellular targets rather than cell surface receptors. Interestingly, there was selective uptake of cNK-2 in the primary monocyte population, whereas cNK-2 was consistently localized inside HD11 cells. Monocytes can differentiate towards macrophages or dendritic cells^39^. The positively stained population suggests differentiation towards macrophages and not dendritic cells, based on the observation of a larger cellular membrane and macrophage-like morphology. The differences in the responses of macrophages and monocytes to cNK-2, although small, are likely attributable to selective interactions with target cells, and macrophage-like cells may be one of the target cells of cNK-2.

In conclusion, we have demonstrated that the chicken cationic peptide cNK-2, as an HDP, has the ability to modulate the innate immune response in macrophages and monocytes through several MAPK pathways and an interaction with target cells. HDPs have been previously associated with protection in several infection models. For example, IDR peptides protect mice against bacterial infection, particularly by multidrug-resistant pathogens, including methicillin-resistant *Staphylococcus aureus*, vancomycin-resistant *Enterococcus, Salmonella enterica* serovar Typhimurium, and *Mycobacterium tuberculosis*, through the induction of chemokines, which results in leukocyte recruitment^24^,^25^,^40^. Based on our findings, the immunomodulatory effect of cNK-2 in avian infection model should be further studied to develop strategies for the prevention of or protection against various infections.

## Materials and Methods

### Peptide and polyclonal antibody synthesis

Four cNK-lysin peptides corresponding to the structural domains of cNK-lysin were synthesized by Genscript peptide services (Genscript, USA) as previously described^12^. The peptides were HPLC purified to >95% purity and confirmed by mass spectrometry. Peptides were generally dissolved to a stock concentration of 2 mg/ml in ultrapure water based on the pre-solubility test result. The resuspended peptides were stored at −80 °C until use and diluted in the appropriate assay medium prior to analysis. Endotoxin contamination of the peptides was evaluated using the Pierce LAL Chromogenic Endotoxin Quantitation Kit (Thermo Fisher Scientific, USA); endotoxin levels were 0.25 EU/ml. The peptide sequences were as follows^12^: cNK-1 (GDDPDEDAINNALNKVCSTG); cNK-2 (RRQRSICKQLLKKLRQQLSDALQNNDD); cNK-3 (VCSTGRRQRSICKQLLKKLRQQ) and cNK-4 (AINNALNKVCSTGRRQRSICKQLLKKLRQQ). To neutralize the activity of cNK-2, rabbit polyclonal cNK-2 anti-sera were developed by Genscript polyclonal antibody services (Genscript), and the polyclonal antibody was purified with protein A agarose (Thermo Fisher Scientific). The specificity of the antibody was confirmed by Western blot (data not shown).

### Antimicrobial assay against *Eimeria* sporozoites

Sporozoites of *Eimeria* spp. were obtained by excystation of sporulated oocysts^11^. Briefly, freshly sporulated oocysts were disrupted with 0.5-mm glass beads for 5–7 s using a Mini-beadbeater (BioSpec Products, USA). The released sporocysts were purified by isopycnic centrifugation in a Percoll gradient, washed in ice-cold Hank’s Balanced Salt Solution (HBSS), and treated with 0.25% trypsin and 0.014 M taurocholic acid (Sigma, USA) at 41 °C for 30 min for *E. acervulina* and 90 min for *E. tenella* to release sporozoites. The sporozoites were collected by filtration, washed 3 times with HBSS at 3000 × *g* for 10 min at 4 °C and resuspended to 1.0 × 10^6^/ml in 10% RPMI-1640 medium (Sigma). The collected sporozoites were incubated with 10 μg/ml melittin (HPLC grade, Sigma) as a positive control^41^ or 30 μg/ml cNK-1, cNK-2, cNK-3 or cNK-4 synthetic peptides at 41 °C for 6 h in RPMI-1640 supplemented with 10% FBS (Invitrogen, USA) and 1% penicillin/streptomycin (Invitrogen). To assess the effect of salt on antimicrobial activity, various concentrations of NaCl (100, 200, or 300 mM) or MgCl_2_ (1, 2, or 3 mM) were added. The parasites were stained with trypan blue, and viable cells were microscopically enumerated.

### Cell culture and cytotoxicity assay

The chicken macrophage cell line HD11^42^ was maintained in complete DMEM supplemented with 10% FBS (HyClone, USA) and penicillin/streptomycin (10,000 unit/ml) (Invitrogen). Chicken primary monocytes were isolated as previously described with modifications^43^. Briefly, peripheral blood mononuclear cells (PBMCs) were isolated from fresh chicken blood collected from healthy birds by a Histopaque (Sigma) density gradient method, and 3 ml of PBMCs were plated into a 6-well plate and incubated at room temperature for 2 h. Non-adherent cells were then removed by triple washing with HBSS. The adherent-enriched monocytes were cultured for 18 h in complete DMEM medium. Prior to stimulation, the cells were washed twice with HBSS and used as primary monocytes. Both cell types were incubated in an atmosphere of 5% CO_2_ at 41 °C with different concentrations of the peptides between 0.1 and 300 μg/ml. The cytotoxic effects of the cNK-lysin peptides were determined using the cell proliferation reagent CCK-8 (Dojindo, USA)^44^, which measures cell viability based on glycolytic production of NAD(P)H. The absorbance was measured at 450 nm, using 650 nm as the reference wavelength.

### Chemokine/cytokine induction assay by qRT-PCR

HD11 cells and primary monocytes were stimulated with various concentrations of the cNK-lysin peptides in a range of 0.1 to 100 μg/ml or with medium alone. RNA was isolated from the HD11 cells or the primary monocytes using the RNeasy Isolation Kit (Qiagen, USA), as per the manufacturer’s instructions, treated with RNase-free DNase (Qiagen) and eluted in RNase-free water (Qiagen). The concentration and purity of the RNA were measured using a NanoDrop spectrophotometer (Thermo Fisher Scientific). cDNA was synthesized using random hexamer primers and a QuantiTect Reverse Transcription Kit (Qiagen). Real-time RT-PCR was performed using a Stratagene Mx3000P (Agilent Technologies, USA) with a QuantiTect SYBR Green PCR Kit (Qiagen) and the various chicken chemokine and cytokine primers listed in Table 1. A melting curve was obtained at the end of each run to verify the presence of a single amplification product without primer dimers. Standard curves were generated using serial, 5-fold dilutions of cDNA. The fold changes in each transcript were normalized to β-actin and are relative to the transcript expression in unstimulated cells (normalized to 1) using the comparative ΔΔCt method as previously described^45^.

### ELISA

The protein levels of chemokines were measured in the cell supernatants by sandwich ELISA using homemade chicken IL-8 antibodies, chCXCLi2 #97 and #100 for IL-8 and ELISA kits from MyBioSource for CCL4 and CCL20.

### LPS-induced inflammatory response

HD11 cells and primary monocytes were stimulated 1 μg/ml lipopolysaccharide (LPS from *E. coli*, 0111:B4) (Sigma). In order to determine if the treatment methods can affect cNK-2 activity, the cells were pre-treated with LPS for 30 min or co-treated with LPS and 10 μg/ml cNK-2. From the preliminary study, we have determined that there was no difference in the expression of IL-1β between two cNK-2 treatment methods. Therefore, cNK-2 may suppress the LPS-induced IL-1β expression through the certain cellular mechanism that does not require the direct interaction with LPS (data not shown). To determine the specificity of cNK-2, an anti-cNK-2 rabbit antibody or normal rabbit IgG (Cell Signaling, USA) were used. As an indicator of inflammation, the expression of chicken IL-1β was determined by qRT-PCR.

### Griess assay

Nitric oxide production in cell culture supernatants was determined by the Griess assay. HD11 cells or primary monocytes were plated into 96-well plates and stimulated with 1 μg/ml LPS, LPS plus 10 μg/ml of cNK-2 or medium alone for 24 h at 41 °C in 5% CO_2_. The collected supernatants were incubated with an equal volume of freshly prepared Griess reagent (Sigma) for 10 min at room temperature, and the absorbance was measured at 540 nm using a microtiter plate reader (BioTek, USA).

### Western blot analysis

HD11 cells and primary monocytes were seeded into 6-well plates and cultured overnight at 41 °C in 5% CO_2_. The cells were incubated for 30 min with cNK-2, 1 μM N-Formyl-Met-Leu-Phe (fMLP) (Sigma) as a positive control, or medium alone. To neutralize cNK-2, an anti-cNK-2 rabbit antibody was added at a concentration of 10 μg/ml. The cell lysates were treated with radioimmunoprecipitation assay buffer, incubated on ice for 30 min and centrifuged to collect the protein samples. The samples were mixed with an equal volume of sample buffer (0.125 M Tris-HCl, pH 6.8, 4% SDS, 20% glycerol, 10% 2-mercaptoethanol, and 0.004% bromophenol blue), heated for 5 min at 95 °C, resolved on a TGX Precast gel (Bio-Rad, USA) and electroblotted onto polyvinyl difluoride membranes using a Trans-Blot Transfer System (Bio-Rad). The membranes were blocked with PBS SuperBlock (Thermo Fisher Scientific) Blocking Buffer for 1 h, washed with 0.1% Tween 20 in PBS (PBS-T), and probed with phospho-p38 MAPK (Thr180/Tyr182) rabbit mAb, phosphor-p44/42 MAPK (ERK1/2) (Thr202/Tyr204) rabbit mAb, phosphor-SAPK/JNK (Thr183, Tyr185) rabbit Ab, or β-actin rabbit Ab (all from Cell Signaling), followed by HRP-conjugated goat anti-rabbit IgG (Thermo Fisher Scientific). The membranes were washed five times with PBS-T, visualized using a Clarity ECL Western Blotting Substrate (Bio-Rad), and detected using the ChemiDoc Imaging System (Bio-Rad).

### Inhibitor and endocytosis assay

To inhibit the MAPK signalling pathway, MAPK-specific inhibitors were used. HD11 cells and primary monocytes were pre-treated with 10 μM SB203580 (p38 inhibitor) (Abcam, USA), PD98059 (ERK inhibitor) (Cell Signaling), SP600125 (JNK inhibitor) (Abcam) or 0.1% DMSO as a vehicle control for 2 h. In a pilot study, we confirmed that the doses of inhibitors used in this study were not toxic to HD11 cells or the primary monocytes. Then, the cells were incubated with 10 μg/ml cNK-2 or medium alone for 4 h. The cell lysates were collected and analysed by qRT-PCR as previously described. To assess the induction of endocytosis by cNK-2, the cells were pre-treated with 5 μg/ml Brefeldin A (Sigma), an endocytosis inhibitor, or medium alone for 1 h prior to stimulation with 10 μg/ml cNK-2.

### Immunocytochemistry

HD11 cells or primary monocytes grown on glass coverslips were stimulated with 10 μg/ml cNK-2 or medium alone for 30 min. The cells were fixed in 4% paraformaldehyde (Sigma) for 10 min, permeabilized in 0.1% Triton X-100 in PBS for 15 min, blocked in SuperBlock (PBS) Blocking Buffer (Thermo Fisher Scientific) containing 2% normal goat serum for 1 h, and incubated with 10 μg/ml cNK-2 rabbit polyclonal antibody for 1 h at room temperature. After washing three times with PBS-T, the cells were incubated with Alexa Fluor 488 goat anti-rabbit IgG secondary antibodies (Thermo Fisher Scientific) for 1 h. After repeated washing with 0.1% Tween 20 in PBS, the cells were stained with Alexa Fluor 555 Phalloidin (Cell Signaling), mounted using Gold Antifade Mountant with DAPI (Thermo Fisher Scientific), and examined using an Eclipse 80i fluorescence microscope (Nikon, Japan), using a fixed shutter speed to permit fluorescence intensity comparison. For confocal microscopic images, a Zeiss 710 confocal laser scanning microscopy (CLSM) system was utilized.

### Statistical analysis

The data were analysed using Student’s t-test or one-way ANOVA followed by Dunnett’s multiple comparison test (GraphPad InStat^®^ software, USA). Differences were considered significant at *p* < 0.05. The data are expressed as the mean value ± the standard error (SE).

## Additional Information

**How to cite this article**: Kim, W. H. *et al*. Evaluation of the Immunomodulatory Activity of the Chicken NK-Lysin-Derived Peptide cNK-2. *Sci. Rep.* **7**, 45099; doi: 10.1038/srep45099 (2017).

**Publisher's note:** Springer Nature remains neutral with regard to jurisdictional claims in published maps and institutional affiliations.

## Supplementary Material

Supplementary Information

## Figures and Tables

**Table 1:** List of quantitative real-time RT-PCR primers used in this study.

Target	Primer and sequence	References
CCL4	(For) 5′-GCTGCCCTTCAGCTTTG-3′	46
	(Rev) 5′-TCAGTTCAGTTCCATCTTGTTCATGTA-3′	
CCL5	(For) 5′-TATTTCTACACCAGCAGCAAATG-3′	47
	(Rev) 5′-GCAGACACCTCAGGTCC-3′	
CCL20	(For) 5′-GAAGGTCATTAAGGGCTT-3′	46
	(Rev) 5′-GGATGTCAATGTGATATGGTTTCC-3′	
IL-8	(For) 5′-GGCTTGCTAGGGGAAATGA-3′	45
	(Rev) 5′-AGCTGACTCTGACTAGGAAACTGT-3′	
IL-1β	(For) 5′-TGGGCATCAAGGGCTACA-3′	45
	(Rev) 5′-TCGGGTTGGTTGGTGATG-3′	
TGF-β1	(For) 5′-CGGGACGGATGAGAAGAAC-3′	45
	(Rev) 5′-CGGCCCACGTAGTAAATGAT-3′	
β-actin	(For) 5′-CACAGATCATGTTTGAGACCTT-3′	45
	(Rev) 5′-CATCACAATACCAGTGGTACG-3′	
