# Supplementary material for: Evaluation of the Immunomodulatory Activity of the Chicken NK-Lysin-Derived Peptide cNK-2
Source: Sci Rep. 2017 Mar 23;7:45099. doi: 10.1038/srep45099 (PMC5362811; doi:10.1038/srep45099)
Supplement: Supplementary Information [file srep45099-s1.pdf]

Supplementary Information

# Evaluation of the Immunomodulatory Activity of the Chicken NK-Lysin-Derived Peptide cNK-2

Woo H. Kim[a], Hyun S. Lillehoj[a], and Wongi Min[b]

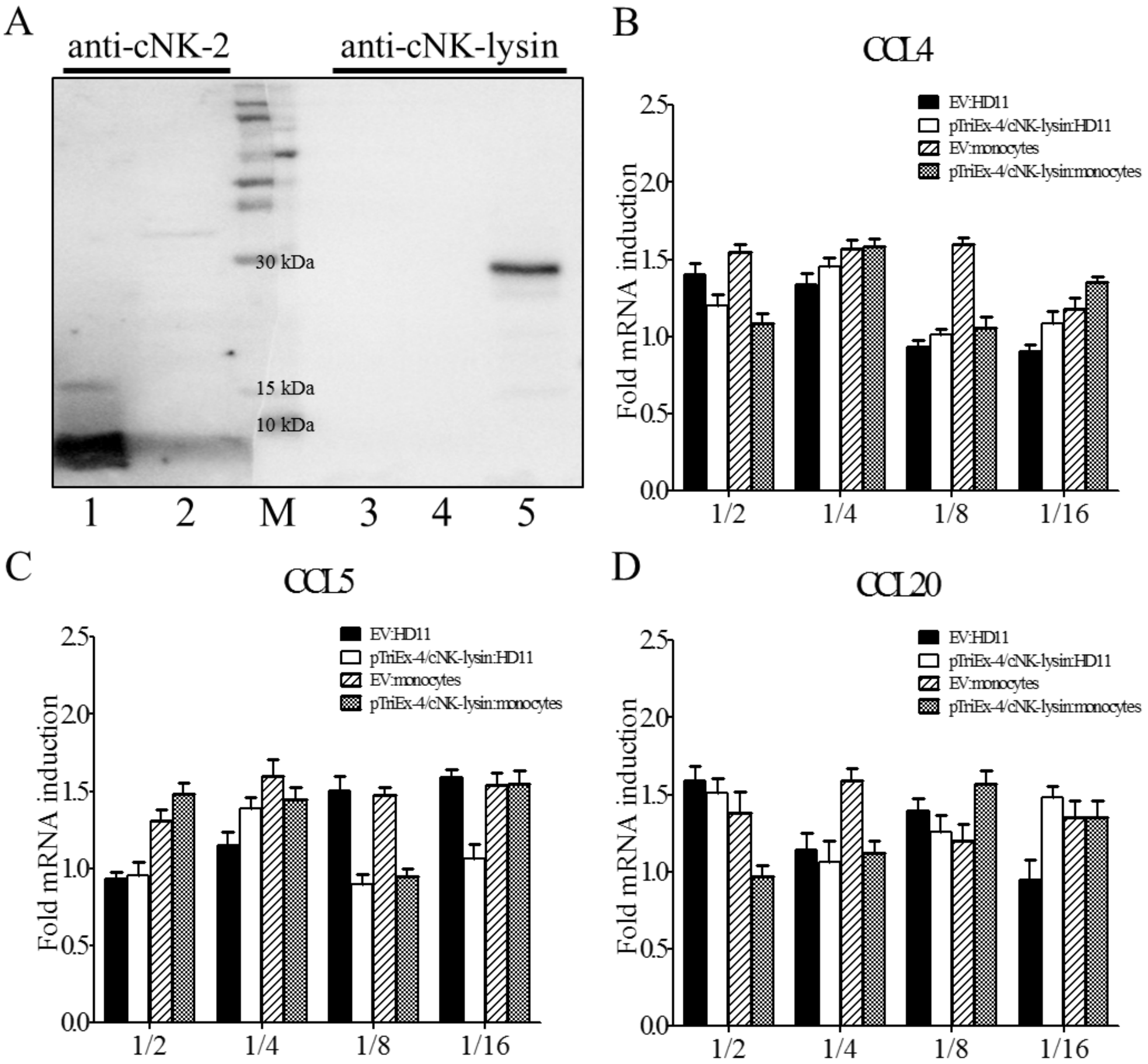


**Supplementary Figure S1. Identification and effect of recombinant cNK-lysin. (A)** COS-7 cells were transfected with TriEx-4/cNK-lysin or empty vector then lysates were blotted with anti-cNK-2 (left) and anti-cNK-lysin poly clonal antibodies. 1 µg of cNK-2 (1 and 3), lysate of COS-7 cells transfected with pTriEx-4/cNK-lysin (2 and 5), and empty vector (4) were loaded and blotted. **(B-D)** HD11 and primary monocytes were stimulated with serial dilutions COS-7 lysate transfected with pTriEx-4/cNK-lysin or empty vector, and analyzed by qRT-PCR.

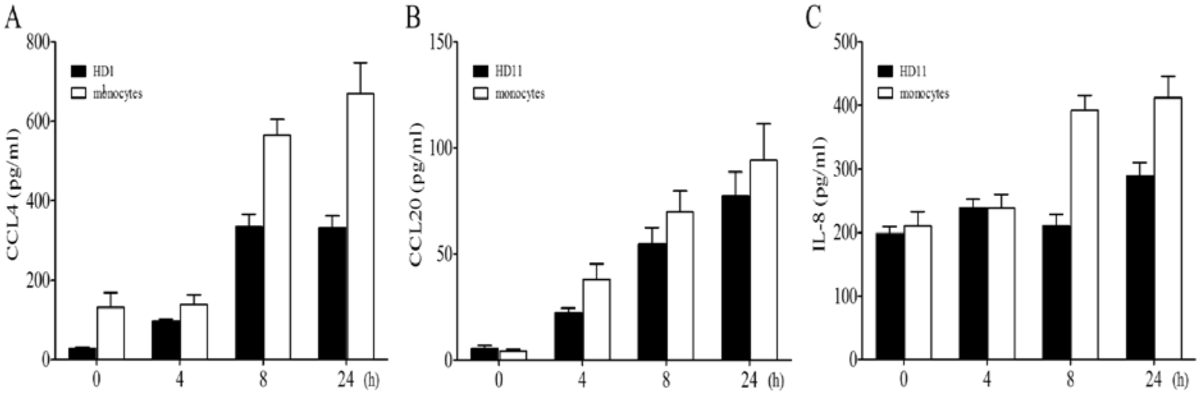


**Supplementary Figure S2. Protein expression of chemokines induced by cNK-2.** HD11 and primary monocytes were stimulated with 10 µg/ml cNK-2 for specified time points. The sandwich ELISA assay was performed for CCL4 (**A**), CCL20 (**B**) and IL-8 (**C**).

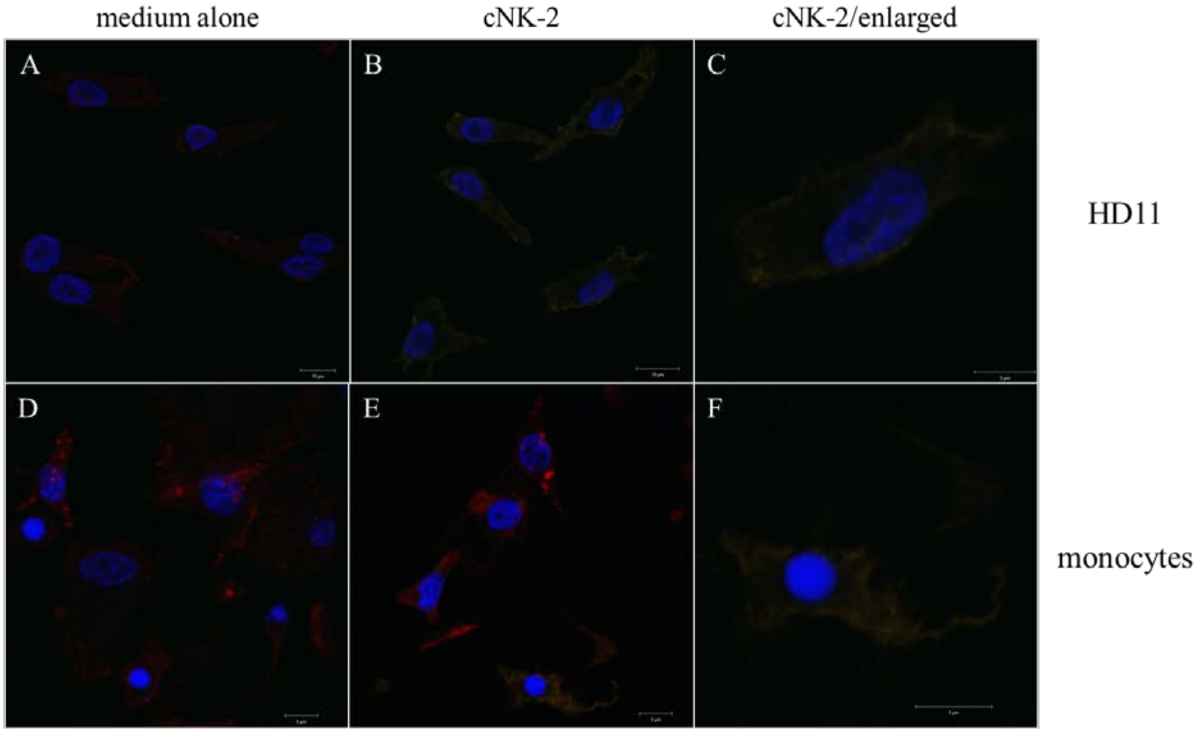


**Supplementary Figure S3. Cellular localization of cNK-2.** HD11 cells (**A**-**C**) and primary monocytes (**D**-**F**) were incubated with medium alone (**A** and **D**) or 10 µg/ml cNK-2 **(B**, **C**, **E** and **F**) for 30 min at 41 °C. Immunocytochemistry was performed with an anti-cNK-2 (green) antibody followed by an Alexa Fluor 488 goat anti-rabbit IgG secondary antibody. DAPI and Alexa Fluor 555 Phalloidin were used to stain nuclei (blue) and F-actin (red), respectively. The images of **C** and **D** are enlarged images of positive cell from **B** and **E**, respectively.
